# Plant terpenes that mediate below‐ground interactions: prospects for bioengineering terpenoids for plant protection

**DOI:** 10.1002/ps.5410

**Published:** 2019-04-08

**Authors:** Ancheng C Huang, Anne Osbourn

**Affiliations:** ^1^ Department of Metabolic Biology, John Innes Centre Norwich Research Park Norwich UK

**Keywords:** terpenes, roots, rhizosphere, plant–microbe, plant–plant and plant–insect interactions, bioengineering, plant protection, root microbiota

## Abstract

Plants are sessile organisms that have evolved various mechanisms to adapt to complex and changing environments. One important feature of plant adaption is the production of specialised metabolites. Terpenes are the largest class of specialised metabolites, with over 80 000 structures reported so far, and they have important ecological functions in plant adaptation. Here, we review the current knowledge on plant terpenes that mediate below‐ground interactions between plants and other organisms, including microbes, herbivores and other plants. The discovery, functions and biosynthesis of these terpenes are discussed, and prospects for bioengineering terpenoids for plant protection are considered. © 2019 The Authors. *Pest Management Science* published by John Wiley & Sons Ltd on behalf of Society of Chemical Industry.

## 1 TERPENES

**Figure 1:**
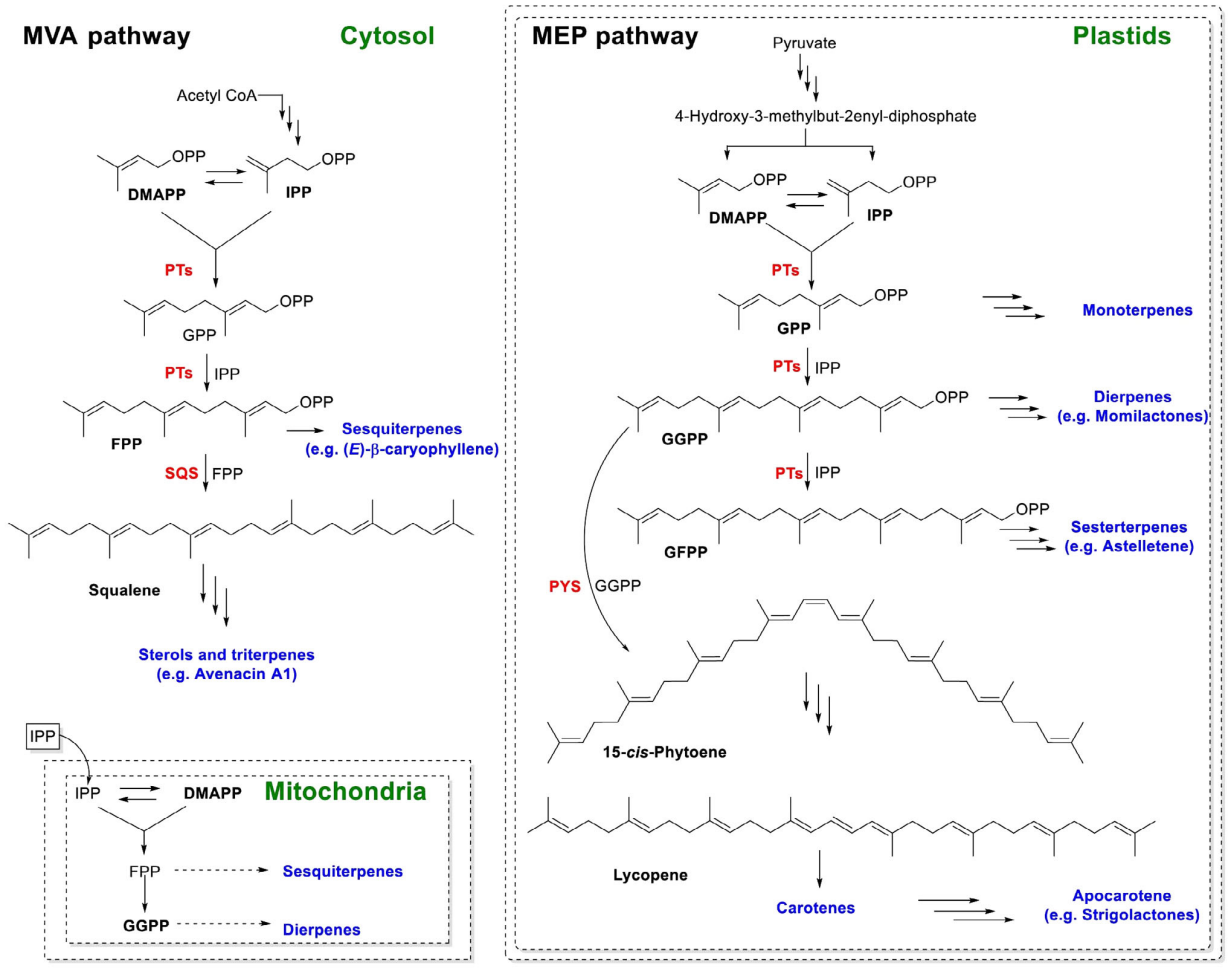
A schematic of the mevalonate and non‐mevalonate pathways leading to the biosynthesis of different terpenes. DMAPP, dimethyl allyl diphosphate; IPP, isopentenyl diphosphate; FPP, farnesyl diphosphate; GGPP, geranylgeranyl diphosphate; GFPP, geranylfarnesyl diphosphate; PTs, prenyl transferases; PYS, phytoene synthase.

**Figure 2:**
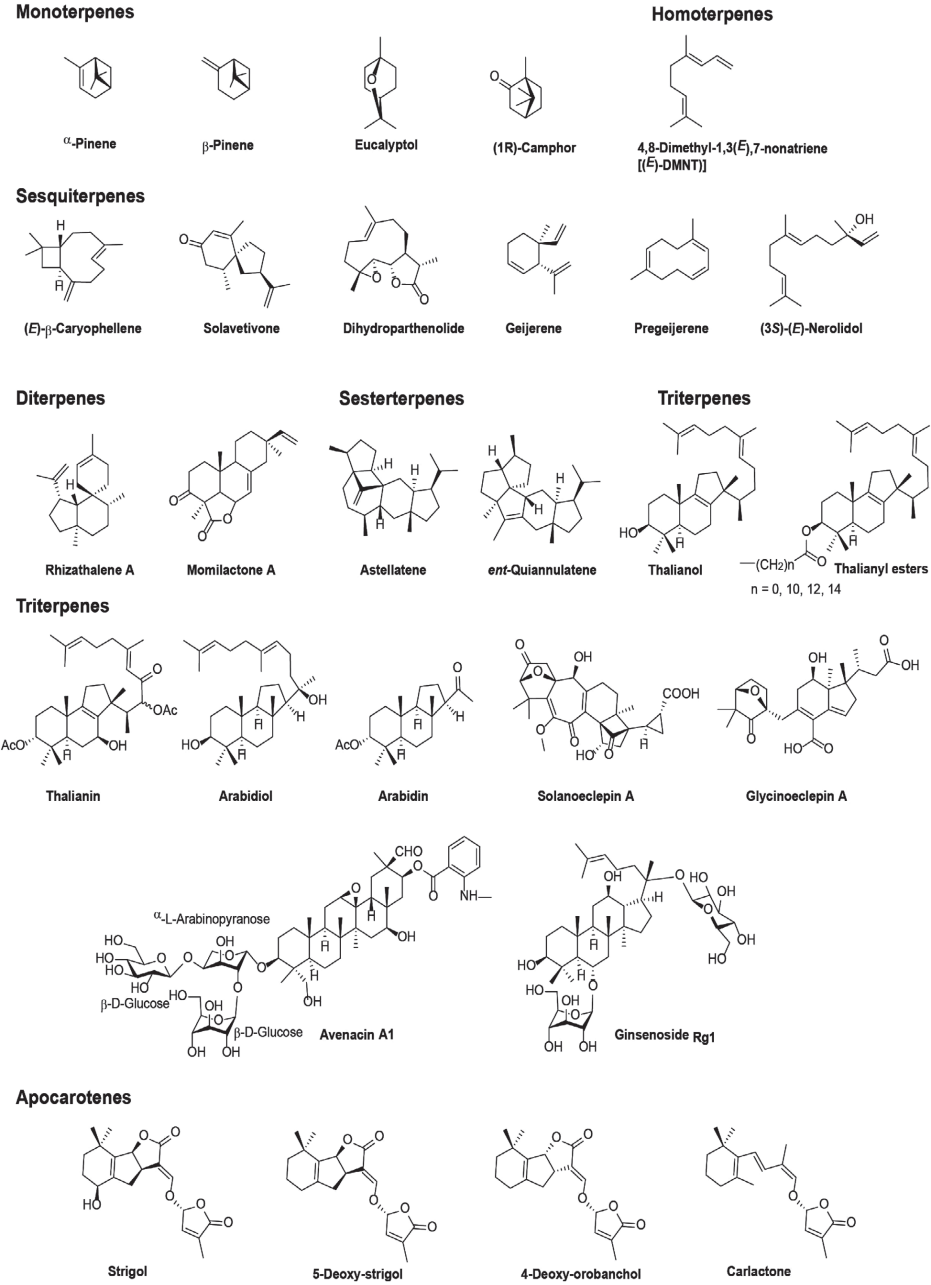
Structures of the different subclasses of terpenes described in this work.

Terpenes are the largest class of natural products, with vast structural and biological diversity.^1^ These compounds consist of five‐carbon isoprene units and can be classified into different subgroups based on the number of these units that they contain, including mono‐terpenes (C10), sesqui‐terpenes (C15), di‐terpenes (C20), sester‐terpenes (C25), tri‐terpenes (C30) and higher terpenes (>C30) such as carotenoids. Terpenes may be biosynthesised via either the mevalonate (MVA) or non‐mevalonate [also known as 2‐*C*‐methyl‐d‐erythritol 4‐phosphate (MEP) or deoxyxylulose 5‐phosphate (DXP)] pathways.^2^, ^3^ Terpene synthases (TPSs)^1^ carry out the first committed biosynthetic step in terpene biosynthesis to generate terpene scaffolds and are present across prokaryotic and eukaryotic organisms, including bacteria,^4^ fungi,^5^ plants^6^, ^7^ and insects.^8^ The terpene scaffolds formed by TPSs can be further modified by tailoring enzymes such as cytochrome P450 monooxygenases (CYPs), acyltransferases and glycosyltranferases for expansion of structural and biological diversity. In higher plants, different types of terpenes are biosynthesised in different compartments. Monoterpenes, diterpenes and sesterterpenes are made in the plastids whereas sesquiterpenes and triterpenes are synthesised in the cytosol (Fig. 1).^3^ Some sesquiterpenes and diterpenes are also synthesised in mitochondria (Fig. 1).^3^ The molecular weights and structures of terpene subgroups determine physical properties such as volatility and influence the way they mediate interactions between different organisms. The roles of volatile terpenes in mediating above‐ground interactions between plants and other living organisms are very well documented.^9^, ^10^, ^11^ For instance, the large amounts of terpenoid volatiles including linalool, farnesene and (*E*)‐nerolidol released by corn seedlings upon feeding by caterpillars help female parasitic wasps [*Cotesia marginiventris* (Cresson)] locate hosts;^11^ the parasitic plant *Cuscuta pentagona* (dodder) also uses volatile cues (mainly terpenoids) from plants such as tomato (*Lycopersicon esculentum*) and wheat (*Triticum aestivum*) for host location.^10^ In comparison, little is known about the nature and roles of terpenes produced by plant roots below the ground. Soils are habitats for an enormous variety of organisms, most notably the bacterial and fungal communities. Macroorganisms (e.g. plants and insects) and microorganisms (e.g. bacteria and fungi) below the ground interact with each other via chemical signals such as small organic molecules. Terpenes can act as an important chemical language that plants use to communicate with other soil‐dwelling organisms. Understanding how different terpenes are biosynthesised and the role that they play in mediating interactions between different organisms can provide a means to manipulate interactions of soil‐dwelling communities, thereby engineering the host plants for health improvement and traits such as pest and disease resistance. Here, we review the roles of volatile and non‐volatile terpenes in mediating below‐ground communications between plants and other organisms (Table 1 and Fig. 2).

**Table 1:** Root terpenes that mediate below‐ground communications

Compounds	Functions	Plants	Reference
β‐Pinene	Induced by the *Diaprepes abbrevatus* larvae; recruits entomopathgenic nematodes (*Steinernema diaprepesi*)	Swingle (*Citrus paradisi*x *Poncirus trifoliata*)	12
Eucalyptol	Attracts cockhafer larve	Oak	13
*E*‐Caryophellene	Attracts an entomopathogenic nematode	Maize	14
Pregeijerene	Induced by the root weevil larvae; recruits entomopathgenic nematodes (*Steinernema diaprepesi*)	Citrus (*Swingle citrumelo*)	15
Geijerene	Induced by the root weevil larvae; recruits entomopathogenic nematodes (*Steinernema diaprepesi*)	Citrus (*Swingle citrumelo*)	15
Solavetivone	Phytoalexin induced by jasmonic acid (JA) and Cu; specific functions unknown	Hairy roots of *Hyoscyamus albus*	16
Rhizathalene A	Confers *Arabidopsis* root resistance to the herbivore fungus gnat (*Bradysia* spp.)	*Arabidopsis*	17
Momilactone A	Phytoalexin against fungi; also has allelopathic effects on lettuce (*Lactuca sativa*)	Rice	18, 19
Dihydroparthenolide	Stimulate germination of *Striga hermonthica*	Common ragweed (*Ambrosia artemisifolia*)	20, 21
Thalianin pathway metabolites	Selectively promote or inhibit root bacteria from different taxa	*A. thaliana*	22
Arabidin	Selectively promote or inhibit root bacteria from different taxa	*A. thaliana*	22
Glycinoeclepin A, B, C	Hatching stimulus for the soybean cyst nematode	Kidney bean	23, 24
Solanoeclepin A	Natural hatching factor of potato and tomato cyst nematodes	Potato and tomato	25
Avenacin A‐1	Antifungal activity against ‘take‐all’ fungus/disease	Oat	26
Ginsenosides	Autotoxic and allelopathic effects	*Panax notoginseng*	27
Strigolactones	Witchweed (*Striga lutea*) germination stimulant; induces hyphal branching in arbuscular mycorrhizal fungi	Cotton	28, 29

## 2 VOLATILE PLANT TERPENES THAT MEDIATE BELOW‐GROUND INTERACTIONS

Terpenes constitute a large proportion of the volatile small molecules that plants produce.^30^ All monoterpenes are highly volatile. The majority of sesquiterpenes are also volatile, with some highly modified ones being semi‐volatile. These volatile organic compounds are capable of travelling long distances via advective transport in gas or aqueous phases or by diffusion, and thus often have roles in long‐range communication or signalling between organisms below ground. The bouquets of volatiles that plants produce have long been believed to be important for plant–plant and plant–insect communications.^9^, ^31^, ^32^, ^33^ However, the bioactive molecules that mediate these interactions have been definitively characterised in only a few cases.

Many insect species have olfactory organs and are thus able to detect the volatiles released by plants to navigate through the soil and reach their host plants. For instance, soil‐dwelling larvae of the forest cockchafer *Melolontha hippocastani* are able to perceive and respond electrophysiologically and behaviourally to the volatiles released by damaged host plant (oak) roots. The monoterpenes eucalyptol and camphor are the major small molecules released by damaged oak roots and can be detected at levels as low as 5 parts per billion volume (ppbv) in soil by larval antennae, so triggering movement of the insect larvae towards the odour source.^13^

In addition to attracting herbivores, volatile terpenes released by damaged roots may also recruit enemies of natural herbivores. The first identified insect‐induced plant signal shown to recruit entomopathogenic nematodes was the sesquiterpene (*E*)‐β‐caryophyllene from maize roots.^14^ Upon feeding by larvae of the Western corn rootworm *Diabrotica virgifera virgifera*, maize roots release (*E*)‐β‐caryophyllene, which attracts significantly more nematodes than other control treatments. Field experiments with authentic (*E*)‐β‐caryophyllene led to a significant decrease in the number of adult *D. v. virgiferea* beetles, supporting the role of (*E*)‐β‐caryophyllene in recruiting entomopathogenic nematodes. A similar phenomenon has also been observed for citrus roots, which release volatiles including the monoterpene β‐pinene and the sesquiterpenes geijerene and pregeijerene in response to infestation by the larvae of the root weevil, *Diaprepes abbreviates*.^12^, ^15^ *In vitro* assays using solvent extracts of weevil‐infested roots and non‐infested roots suggest that the former is more attractive to nematodes, supporting a role of these volatiles in recruiting nematodes. The same research group further showed that such below‐ground volatiles were produced by roots challenged by soil insects but not by other above‐ground herbivores, nor were they produced by the shoots of the host plants, suggesting site‐specific induction and biosynthesis of these volatiles in roots.

## 3 NON‐VOLATILE TERPENES THAT MEDIATE BELOW‐GROUND INTERACTIONS

Diterpenes (C20), sesterterpenes (C25), triterpenes (C30) and other larger terpenes (>C30) have higher molecular weights and are thus heavier and not volatile (although some diterpenes might be classified as semi‐volatile). Diterpenes and sesterterpenes are biosynthesised in the plastids and triterpenes in the cytosol. Some of these non‐volatile terpenes serve as phytoalexins that are produced only in response to pest, pathogen or elicitor challenge whereas others are constitutively synthesised as part of normal growth and development. Many of these non‐volatile root terpenes are exuded from plant roots,^18^, ^28^, ^34^ where they serve as the first line of plant defence and mediate rhizosphere community establishment. Due to their low volatility, some compounds exuded by plants can be deposited in the soil and confer long‐lasting impact on the soil‐dwelling communities.^27^

The diterpene momilactones A and B were first isolated from the seed husk of rice and reported to inhibit the growth of rice roots at less than 100 ppm.^35^ Momilactones are also phytoalexins produced by rice in response to pathogen attack or when elicited with chitin oligosaccharide, components of fungal cell wall that mimic fungal attacks.^19^ Momilactone A can be detected in rice root exudates and genetic knockout of the diterpene synthase gene involved in the first committed step for the synthesis of this compound resulted in compromised allelopathic effects towards the roots of co‐germinated neighbouring lettuce seedlings, supporting a role of momilactones in allelopathy.^18^ Another diterpene that is known to be involved in defence against root herbivores is rhizathalene, which is synthesised in the root leucoplasts (non‐pigmented plastids) of the model plant *Arabidopsis thaliana*. *A. thaliana* mutants lacking rhizathalene are more susceptible to the opportunistic root herbivore fungus gnat (*Bradysia* spp.) and suffer substantial damage of peripheral tissue at the larval feeding sites. Apart from diterpenes, *A. thaliana* roots also produce other terpenes, including sesterterpenes (e.g. astellatene, a relatively rare subclass of terpenes in terms of the number of structure entities isolated from nature)^36^ and triterpenes (thalianol, marneral, arabidiol and their derivatives).^22^, ^37^, ^38^, ^39^, ^40^ Although the sesterterpenes produced by *A. thaliana* roots structurally resemble fungal sesterterpenes, with *ent*‐quiannulatene being the enantiomer of quiannulatene produced by the fungus *Emericella variecolor*, it is still not yet clear whether these *A. thaliana* sesterterpenes are involved in mediating below‐ground interactions. In comparison, the role of triterpenes produced by *A. thaliana* roots in mediating below‐ground communications is more evident. The cleavage product of arabidiol, homoterpene (*E*)‐4,8‐dimethyl‐1,3,7‐nonatriene (DMNT), has been implicated in defence against the root rot pathogen *Pythium irregulare*.^37^ Root triterpenes thalianin, thalianyl medium chain fatty acid esters, arabidin and their pathway intermediates have recently been identified and shown to be able to directly modulate *A. thaliana*‐specific root bacterial communities in a very selective fashion.^22^ Moreover, some root bacteria were found to be able to selectively metabolize certain triterpenes derived from these pathways and utilize the breakdown products as carbon sources for proliferation.^22^ These findings suggest that root triterpenes are indeed actively involved in shaping an *A. thaliana*‐specific root microbiota.

The activities of triterpenoids in mediating below‐ground interactions have also been revealed in crop plants as well as in *A. thaliana*. For instance, glycinoeclepin A, a triterpenoid first isolated from kidney bean in 1982, can stimulate hatching of larvae from the eggs of cyst nematodes (genera *Heterodera*, *Globodera* and others) *in vitro* at a concentration of 10^−11^ to 10^−12^ g mL^−1^ in water at 25 °C.^23^, ^41^ Following the isolation of glycinoeclepin A, another hatching stimulus for cyst nematodes of *Globoder rostochiensis* and *Globodera pallida* was isolated from potato and structurally established as solanoeclepin A, a triterpenoid that structurally resembles glycinoeclepin A.^25^

Besides triterpene aglycones, glycosylated triterpenes also mediate below‐ground interactions. Avenacins A‐1, A‐2, B‐1 and B‐2 are antifungal triterpene glycosides that protect oats from ‘take‐all’, a soil‐borne fungal disease that causes major yield losses on wheat.^26^ Oat mutants that are unable to make avenacin A‐1 are compromised in their resistance to ‘take‐all’ and other fungal pathogens.^42^ Since oat roots produce avenacins and are resistant to ‘take‐all’, oat can be cultivated in soils infected by ‘take‐all’ fungus as a break crop prior to planting wheat. Besides antifungal activity, comparative metatranscriptomics of the rhizosphere microbiome of different plants, including oat and avenacin‐deficient oat mutants, suggest that avenacins may have a broader role in impacting the eukaryotic community.^43^ Another example of triterpene glycosides affecting the eukaryotic organisms are the allelopathic effects of autotoxic ginsenosides produced by *Panax notoginseng*.^27^ Soils cultivated with *P. notoginseng* can often result in replant failure.^27^

There are very limited examples of higher terpenes (>30) that mediate below‐ground interactions, the best characterized being the apocarotenoid strigolactones.^44^ Strigol was the first strigolactone to be reported.^28^ This compound was isolated from cotton root exudates in 1966 and found to be a potent stimulant of the germination of witchweed (*Striga lutea Lour*.) seeds, with activity detected at <10^−5^ ppm.^28^ Strigolactones were mistakenly regarded as sesquiterpene lactones originally. Interestingly, some sesquiterpene lactones such as dihydroparthenolide that are structurally reminiscent of strigolactones have been isolated from common ragweed, *Ambrosia artemisiifolia*, and also found to have similar stimulation effects for witchweed germination.^20^, ^21^ The deoxy form of strigol, 5‐deoxy‐strigol, was later isolated from root exudates of *Lotus japonicus* in 2005 and found to induce extensive hyphal branching in germinating spores of the arbuscular mycorrhizal (AM) fungus *Gigaspora margarita* at very low concentration (100 ng–30 pg per disk using the diffusion assay).^29^ Strigolactones are now regarded as an important class of plant hormones since not only can they mediate communication between parasitic plants and AM fungi, but they have also been shown to inhibit shoot branching in plants such as garden pea (*Pisum sativum* L.) and have diverse roles in plant development.^45^, ^46^ The wide spectrum of activity of strigolactones has attracted substantial research efforts on elucidating their biosynthesis and functions.^44^, ^46^, ^47^ The multifaceted functions of strigolactones also demonstrate the important roles of small molecules in plant adaptation to natural environments.

## 4 BIOSYNTHESIS OF THE BIOACTIVE ROOT TERPENES

The biosynthesis of volatile mono‐ and sesquiterpenes described in this work is relatively simple, involving only TPSs that fold the precursors geranyl diphosphate (GPP) and farnesyl diphosphate (FPP) into different scaffolds (Fig. 1). The TPSs that catalyse the formation of monoterpenes α‐pinene, β‐pinene, and eucalyptol and sesquiterpene (*E*)‐β‐caryophyllene have been identified and characterised from loblolly pine (*Pinus taeda*), sweet wormwood (*Artemisia annua*), garden sage (*Salvia officinalis*) and maize (*Zea mays*), respectively,^48^, ^49^ whereas the biosynthesis of sesquiterpenes geijerene and pregeijerene still remains elusive.

The majority of the non‐volatile terpenes described in the previous section are highly modified compounds that require multiple genes for their biosynthesis, except for rhizathalenes,^17^ astellatene^36^ and *ent*‐quiannulatene,^36^ which can be synthesised by one single root‐expressed *A. thaliana* TPS from their corresponding diphosphate precursors (Fig. 1). The biosynthetic pathways for triterpenes thalianin and arabidin from *A. thaliana* have recently been elucidated. Biosynthesis of thalianin involves seven enzymes acting sequentially after the formation of the universal triterpene precursor 2,3‐oxidosqualene, including a oxidosqualene cyclase (THAS),^39^ two CYPs (THAH, THAO) that introduce the C7 β‐OH, C15‐OH and C16 = O, two BAHD acyltransferases (THAA1, THAA2) that install acetyl groups onto the C15 and C3‐OH, respectively, and two Rossman‐fold alcohol dehydrogenases/oxidoreductases (THAR1, THAR2) that epimerise the C3‐OH. All the biosynthetic genes in the thalianin pathway have been identified and functionally characterised both in heterologous host and *in vivo*.^22^ The missing genes in the arabidin pathway have also been identified by the same authors. Interestingly, the thalianin and arabidin pathways are divergent pathways that share one common acyltransferase (THAA2) and one alcohol dehydrogenase (THAR2) gene.^22^ One membrane‐bound *O*‐acyltransferase gene (*THAA3*) has also been found to be involved in the biosynthesis of thalianyl medium‐chain fatty acid esters in *A. thaliana* roots, although this gene appeared to be partially redundant in *A. thaliana*.^22^ Apart from the root triterpenes in *A. thaliana*, the biosynthetic pathway for avenacin A1 in oat has also been studied extensively over the past decades and many pathway genes have been identified using a forward genetic approach.^42^, ^50^, ^51^, ^52^, ^53^ A few genes have also been biochemically characterised.^53^, ^54^, ^55^ These include genes that encode an oxidosqualene cyclase (saponin deficient 1, SAD1) that synthesises the β‐amyrin scaffold,^53^ a multifunctional CYP(SAD2) that oxidizes β‐amyrin to install a C_12−_C_13_ epoxide and a C_16_ hydroxyl group,^54^ another CYP (SAD6) that introduces the C_21_ hydroxyl group,^56^ a methyl transferase (SAD9) that methylates anthranilate,^55^ a glucosyltransferase UGT74H5 (SAD10) that glucosylates *N*‐methyl anthranilate,^51^ a serine carboxypeptidase‐like acyltransferase (SCPL, SAD7) that acylates the deacyl avenacins,^55^ and an arabinosyltransferase (UGT99D1) that catalyses the addition of an l‐arabinose to the triterpene scaffold at the C3 position.^57^ The biosynthesis of ginsenosides is under extensive investigation due to the medicinal properties of these compounds. Many genes involved in the biosynthesis of various ginsenosides have been identified, although the complete biosynthetic pathways are yet to be fully elucidated.^58^ Readers are referred to the recent review that summarises the current knowledge regarding ginsenoside biosynthesis.^58^ Like avenacins and ginsenosides, the biosynthesis of strigolactones is also partially elucidated.^47^ Strigolactones are derived from carotenoids and many genes/enzymes involved in converting carotenoids to intermediate carlactone and further modified product 4‐deoxy‐orobanchol have been identified (Figs 1 and 2).^47^, ^59^, ^60^ However, there are still some enzymes missing in the biosynthesis towards strigol. Other highly modified triterpenes such as glycinoeclepin A and solanoeclepin A have been chemically synthesised and structures confirmed.^24^, ^61^ However, little is known about their biosynthesis at present.

An intriguing phenomenon in the biosynthesis of some of the terpenes described here is that the biosynthetic genes in some pathways are physically clustered to form biosynthetic gene clusters in the respective plant genomes (e.g. those required for the synthesis of diterpene momilactone A^19^ and triterpenes thalianin,^22^, ^39^ arabidin^22^, ^37^ and avenacins^62^) whereas others are not (e.g. those for the biosynthesis of carotenoids and apocarotenoids such as strigolactones).^63^ It is still unclear why some biosynthetic genes are clustered in plant genomes. However, gene clustering is likely to be a consequence of strong selection pressure to confer important functions to plants (e.g. ecological advantages) from an evolutionary perspective.^39^, ^63^, ^64^ The ecological benefits of gene clustering are demonstrated to a certain degree by the aforementioned terpenes (e.g. momilactone A, avenacin A‐1, arabidiol/DMNT) in defending plants against other soil‐dwelling organisms and in directing the assembly of the *A. thaliana*‐specific root microbiota (thalianin). The clustering of biosynthetic genes might also be driven by the need for more efficient synthesis and regulation of natural products in plants, an art crafted by nature for important traits in plants. We are learning from nature by assembling individual biosynthetic pathway genes into an integrate genetic cassette for bioengineering the synthesis of bioactive natural products in different plants for desired agronomically important traits.

## 5 BIOENGINEERING TERPENES FOR BELOW‐GROUND PEST MANAGEMENT

Understanding the molecular basis for the biosynthesis of bioactive terpenes is the prerequisite for bioengineering terpenes. Enzymes involved in the MVA and MEP pathways are well characterised. In contrast, knowledge of the biosynthetic enzymes that catalyse the formation of specialised terpenoids is far more fragmented. Finding all the biosynthetic genes for pathways that confer an agronomic advantage is the first step towards precise metabolic bioengineering for crop protection. There are several strategies for bioengineering terpenes for pest management and crop protection. One can either genetically manipulate the target crop plants for terpenoid production via stable transformation or engineer heterologous hosts for producing terpenes for exogenous chemical application. Genetic modification of the target plant is an attractive strategy since, once introduced, the trait can be inherited naturally over generations, and the genetically engineered materials can also be used for breeding to introduce desired traits to other crop varieties susceptible to pests, although this also comes with concerns of threats to the environment,^65^, ^66^ and potential undesired detrimental physiological and ecological impacts on the genetically modified plants. An alternative solution is to engineer heterologous hosts for the bioproduction of small molecules. Compared to genetically modifying plants, heterologous production requires less regulation but demands extensive effort for process development.

Some attempts have been made to engineer plant volatiles for pest management. The sesquiterpene (*E*)‐β‐caryophyllene synthase gene from oregano has been engineered into wild and cultivated maize.^67^, ^68^ This resulted in constitutive emission of the volatile (*E*)‐β‐caryophyllene. Field experiments in rootworm‐infested plots showed that the genetically modified plants that emitted (*E*)‐β‐caryophyllene suffered significantly less root damage with 60% fewer adult beetles (adult stage of western corn rootworm) present than the non‐(*E*)‐β‐caryophyllene‐emitting plants.^69^ However, (*E*)‐β‐caryophyllene is a signalling molecule that also attracts above‐ground herbivores, including the pest *Spodoptera frugiperda*. Overexpression of the terpene synthase gene constitutively under the control of a maize ubiquitin promoter resulted in (*E*)‐β‐caryophyllene production above ground and led to increased leaf damage by herbivores and compromised seed germination, plant growth and yield, although the roots did not suffer more damage, possibly due to the recruitment of the entomopathogenic nematodes.^67^, ^68^

Another sesquiterpene that has been engineered for plant protection is the (3*S*)‐(*E*)‐nerolidol, one constituent of the herbivore‐induced volatile blend of maize and tomato.^70^ By targeting a nerolidol synthase gene from strawberry for expression in the mitochondria of *A. thaliana* (as opposed to the conventional sesquiterpene biosynthesis compartment cytosol), an elevated level of production of (3*S*)‐(*E*)‐nerolidol was achieved together with the formation of (*E*)‐DMNT via side‐chain cleavage by an unknown endogenous cytochrome P450 oxidase in *A. thaliana*. Interestingly, the engineered plants attracted significantly more ‘bodyguard’ predatory mites (enemies of plant herbivores) than the undamaged wild‐type *A. thaliana* plants without any significant impact on plant fitness. Only slight retardation of the growth of basal rosette of the engineered plants was observed, suggesting the feasibility of engineering terpenoids for plant protection.^70^

The two aforementioned examples both used constitutive promoters, which resulted in overexpression of the compounds all over the plants. Such engineering is semi‐targeted/unprecise as it aims to increase the production of specific molecules but without targeting their biosynthesis to the site of action, which could potentially create undesired side effects. Targeted/precision engineering is warranted to alleviate/overcome possible undesired impacts. This would involve choosing specific genes for the biosynthesis of particular terpenes and engineering them to be expressed under specific promoters at the site of action in plants (e.g. specific tissues or cell types), rather than constitutive expression. For instance, the promoter of the β‐amyrin synthase gene from oat works in other plant species (e.g. *A. thaliana*, rice and *Medicago truncatula*), so enabling expression specifically in the epidermal cells of the root tips.^71^ This also means that it is possible to engineer the avenacin pathway/other antimicrobial triterpenes into other plant species (e.g. wheat) to tackle diseases such as ‘take‐all’. Promoters as such are important tools towards precise engineering.

Another rapidly expanding and evolving technology that enables precise genetic manipulation is the clustered regularly interspaced short palindromic repeats (CRISPR)/CRISPR‐associated protein 9 (Cas9) system, known as CRISPR/Cas9.^72^, ^73^ This system originated from the bacterial immune mechanism for clearance of foreign invading DNAs from phages, and has now been developed to allow for precise gene‐editing in virtually every genome.^74^ The mechanisms and applications of CRISPR/Cas9 have been reviewed in detail and readers are referred to these reviews for further details.^74^, ^75^ CRISPR/Cas9 has been widely used in plant genome editing.^76^ One can either knock‐out, creating mutations on targeted genes related to diseases,^77^ or knock‐in and replace genes at specific loci with genes of interest to introduce desired traits.^78^ The rapid development and maturation of this technique will greatly accelerate the precise engineering of terpene biosynthesis in plants for pest and disease resistance.

In contrast to genetically modifying plants, direct application of chemicals (e.g. (*E*)‐β‐caryophyllene)^14^ into the soil has been used conventionally in managing pests. Production of terpenes at scale for exogenous application as crop protection chemicals requires either chemical synthesis or bio‐production in heterologous hosts. Production of structurally complex terpenes via chemical synthesis is almost infeasible in most cases considering the extremely poor benefit‐cost ratio.^79^ In contrast, bio‐production of terpenes in heterologous hosts can be achieved at scale and in a sustainable fashion.^79^ Various organisms have been tested as heterologous hosts for bio‐production of plant natural products such as terpenes. Prokaryotic microbial organisms such as *Escherichia coli* have been studied extensively as platforms for microbial engineering of natural product pathways. The biggest advantage of bacterial systems is their fast proliferation rate. *E. coli* is an excellent organism for engineering production of terpene hydrocarbon scaffolds.^80^, ^81^, ^82^, ^83^ However, the functionalisation of these scaffolds is challenging since *E. coli* lacks the necessary cytochrome P450 reductases and endoplasmic reticulum structure to support expression of plant P450s.^84^, ^85^ Although progresses in engineering plant P450s for terpenoid biosynthesis have been made,^80^, ^86^, ^87^ the challenges of engineering multiple P450s together with other types of enzymes in *E. coli* remain serious and unaddressed.

Yeast is a popular eukaryotic organism for heterologous production of terpenes. Many different strategies have been developed for engineering yeast for terpenoid production.^88^, ^89^, ^90^ One can engineer the upstream pathway to increase the overall precursor supply, block the downstream pathway to shuffle metabolic flux towards production of desired terpene classes, or make use of the different subcellular compartments for enhanced biosynthesis of terpenes. Yeast is a versatile and powerful engineering host for production of terpenoids as it can accommodate many different types of enzymes,^91^ albeit enormous efforts are required to optimise and generate strains with improved titres. For example, engineering of high‐level yeast production of artemisininic acid, a precursor for the anti‐malarial drug artemisinin, is reported to have taken 150 person years.^92^ Progress in engineering yeast for terpene production has been summarised in a recent review.^93^

Besides microbes, plants can be excellent heterologous hosts for the production of terpenes. The photosynthetic nature of green plants means that the carbon source directly originates from atmospheric CO_2_ and no other exogeneous carbon supply is required, which is in stark contrast to microbial hosts such as *E*. *coli* and yeast. Another advantage of plants as a bioproduction factories is the availability of universal substrates, enzymes and cofactors in primary metabolism and organelles present in plant cells for bioengineering enzymes of plant origins. Powerful new expression technology also opens up unprecedented opportunities to rapidly characterise plant natural product biosynthetic enzymes and pathways, and achieve elevated production of small molecules in plants such as *Nicotiana benthamiana*, a wild relative of tobacco. *N. benthamiana* is an excellent heterologous host for terpenoid production.^94^ We recently demonstrated the capacity of engineered *N. benthamiana* for producing gram‐scale amounts of terpenoids.^94^, ^95^, ^96^, ^97^ By engineering upstream terpene pathway enzymes (e.g. a feedback‐insensitive HMG CoA reductase) and TPSs into a hyper‐translation expression vector (pEAQ) together with a custom‐designed large‐scale vacuum infiltration device, rapid high‐level production of various terpenoids was achieved. Such a platform requires relatively less engineering effort and is easy to use. It is also noteworthy that once an engineered pathway is optimised using transient expression in *N. benthamiana*, transgenic *N. benthamiana* that is stably transformed with the optimised engineering pathway cassettes can be generated to achieve continuous production of desired molecules. *N. benthamiana* is a representative species and such an approach may be applied to other plant species that are fast‐grown, efficient in biosynthesis and have high biomass.

## 6 CONCLUSIONS AND PERSPECTIVE

As the largest class of natural products, terpenoids have been very well studied. However, our current knowledge of the biosynthesis and roles of terpenes produced by plant roots is still very limited. The remarkable chemical diversity of plant roots is largely untapped, given the presence of numerous uncharacterised genes with predicted functions in plant natural product biosynthesis in plant genomes, a considerable number of which may be root‐expressed. The complex nature of root metabolites, many of which are unknown molecules of low abundance and produced only when elicited under certain conditions, present challenges in elucidating their composition and functions. Furthermore, we also know very little about the spatial distribution of metabolites in plant roots, how they are transported and exuded, and the mechanisms by which they interact with other soil‐dwelling organisms. Apart from the many unanswered questions regarding the biosynthesis of small molecules in plant roots, the enormous diversity of soil‐dwelling organisms is another ‘dark matter’ that remains largely unexplored. Plant microbiota contains countless bacteria and fungi that have evolved to coinhabit inside (endophytes) and outside (rhizosphere) plant roots and are key players in the multi‐component ecosystems that shape plant health.^98^, ^99^ Previous studies on root microbiota have generated significant insights into the composition of plant microbiota,^100^, ^101^ however, with the development of technologies and methodologies in microbiome research, the focus is now shifting from phenotype‐based to more causation‐ and mechanism‐driven research.^102^ Understanding what the beneficial and detrimental/pathological components are as well as the mechanisms that would allow manipulation of the behaviours/ratios/interactions of these microbial members is a prerequisite for engineering plant microbiota for biocontrol and improvement of plant growth.^98^ Understanding the biology of different organisms in the interaction will also yield mechanistic insights and allow for more targeted manipulations. Small molecules produced by plant roots are one form of output that can both directly and indirectly mediate interactions between plants and other organisms.^103^, ^104^ Knowledge of the molecular basis of small molecule biosynthesis and transport, coupled with understanding of the composition and causation of the establishment of root microbiota and other macro‐organism associations will together provide new solutions to engineering plants for pest and disease resistance in the future.
